# Case Report: The use of a 3D printed alignment guide system for correction of a delayed union/nonunion/malunion femoral fracture in a dog

**DOI:** 10.3389/fvets.2025.1562071

**Published:** 2025-07-04

**Authors:** Radu Scortea, Cosmin Muresan, Maximiljan Krauß

**Affiliations:** ^1^Department of Surgery, AniCura Tierklinik Düsseldorf, Düsseldorf, Germany; ^2^Faculty of Veterinary Medicine, Department of Surgery, Anesthesia and Intensive Care, University of Agricultural Sciences and Veterinary Medicine of Cluj-Napoca, Cluj-Napoca, Romania

**Keywords:** delayed union, nonunion, femoral fracture, 3D-printed alignment guide, trauma

## Abstract

A two-year-old neutered male mixed-breed dog was presented with a history of chronic lameness of the right pelvic limb. Physical examination and diagnostic imaging report revealed a delayed union with suspected progression toward nonunion/malunion of the femur. Further assessment showed the presence of a multiplanar deformity with a distal femoral valgus, a caudomedial translation of 26 mm, a femoral torsion angle of 35° compared to 21° to the left, and an excessive limb shortening of 34% in the frontal plane/ 31% in the sagittal plane. A 3D-printed alignment guide (3DPAG) was designed, printed, and successfully used to reduce the delayed union/nonunion fracture. The pre-contoured LCP plate, secured with seven screws, provided stable fracture fixation. The 3DPAG contributed significantly to achieving accurate alignment and implant placement.

## Introduction

Nonunion, delayed union, and malunion are terms currently used to describe abnormal bone fracture healing outcomes. Nonunions are seen in the absence of radiographic evidence of fracture healing regardless of time, while delayed unions occur when a lengthening of the healing process beyond the expected timeframe is noted (1, 2). Delayed unions can lead to malunions, where bones heal with an abnormal anatomical alignment (1, 2). Evaluating chronic fractures and angular limb deformities with radiographs may not always be accurate, and therefore, obtaining a computed tomography (CT) is preferred (3–6). Pre-operative virtual planning and 3D printing for correcting angular limb deformities (ALDs) have become popular in veterinary medicine, facilitating reduction and correct alignment of fracture segments (5, 7–9).

Although two recent case reports in dogs describe the employment of additive manufacturing for the correction of a femoral malunion with an interlocking nail using 3D-printed osteotomy and reaming guides (10) and the repair of a femoral non-union with a 3D-printed titanium bio-scaffold, rh-BMP, and double plating (11), their approach differs in concept and technique. Therefore, this case report describes the reduction and stabilization of a femoral delayed union/nonunion/malunion fracture in a dog using a 3D-printed alignment guide (3DPAG) in conjunction with a plate and screws.

## Case presentation

**Figure 1:**
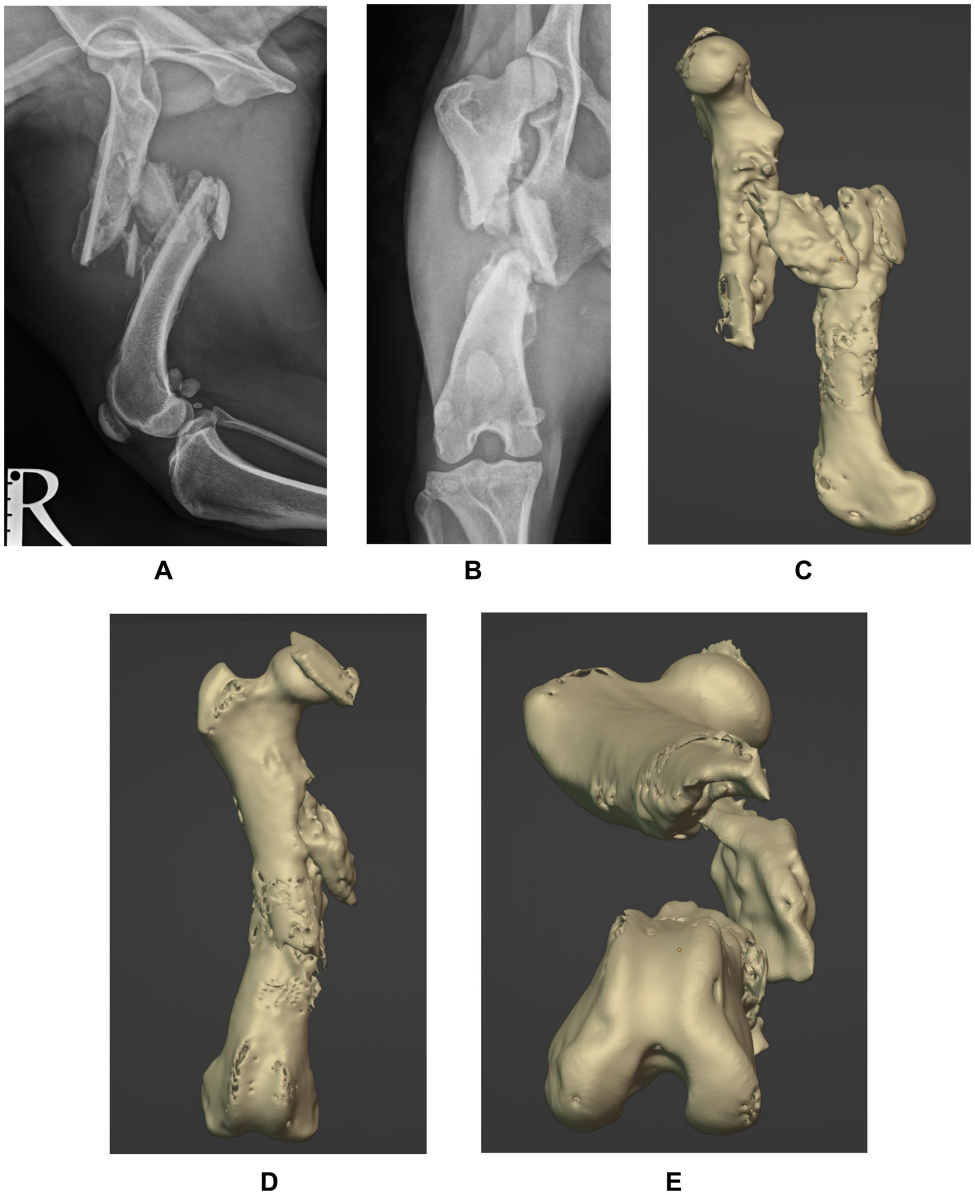
Preoperative mediolateral **(A)** and caudocranial **(B)** radiographs of the right femur and 3D reconstructions of the sagittal **(C)**, frontal **(D)** and transverse **(E)** planes showing a chronic, closed, highly-comminuted, mid-diaphyseal, non-reconstructable fracture of the right femur, with caudo-medial displacement. Note callus involving the mid-diaphysis and the excessive periosteal reaction at the fracture site.

A two-year-old rescue dog, mixed-breed, neutered male, weighing 14.2 kg, was presented with a history of chronic lameness of the right pelvic limb. The exact date of the presumed trauma was unknown, as the dog showed signs of lameness since adoption (five weeks before assessment). Orthopedic examination revealed a severe weight-bearing lameness of the right pelvic limb, muscular atrophy, pain, and crepitus on manipulation. Orthogonal radiographs confirmed the presence of a chronic, closed, highly-comminuted, mid-diaphyseal, non-reconstructable fracture of the right femur, with caudo-medial displacement (Figures 1A,B), with vicious callus involving the mid-diaphysis and periosteal new bone formation extending along the proximal and distal diaphysis. Due to the unknown date of fracture onset and radiographic findings, the lesion was considered a delayed union, progressing to nonunion/malunion if not appropriately stabilized. A CT of both pelvic limbs was performed using a Siemens Healthcare Somatom (64-slice) with the patient positioned in ventral recumbency and imaging parameters set at 120 KV, 200 mAS, and a slice thickness of 0.6 mm. 3D reconstructions were performed using a bone-specific algorithm.

**Figure 2:**
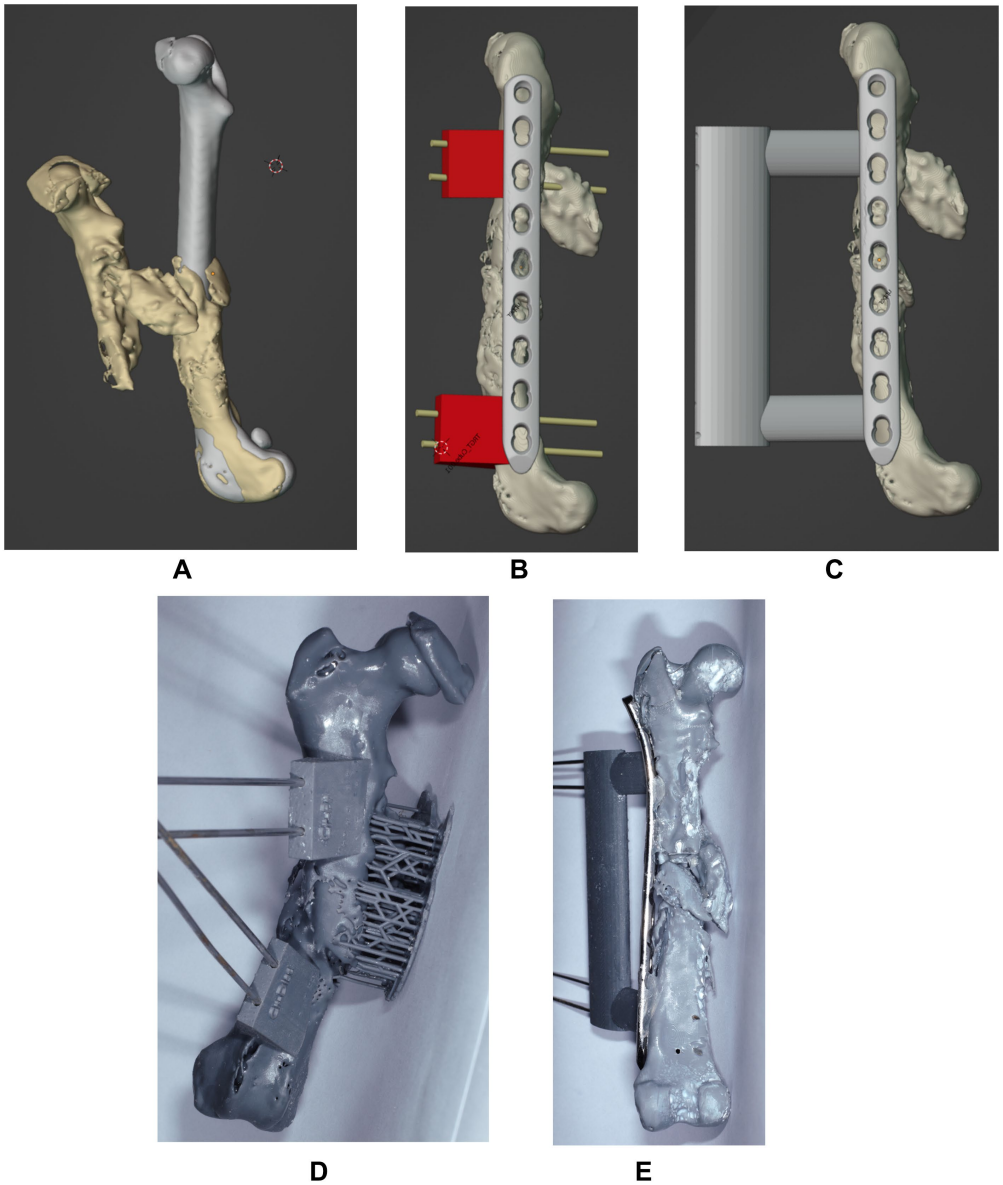
Sagittal projection of the mirrored femur used as a model and superimposed with the affected right femur for correction **(A)**. Projection of the virtually aligned femoral model with OG and a 3.5 mm LCP plate **(B)** and of the virtually aligned femoral model with 3DPAG and plate **(C)** in sagittal plane. Craniocaudal image **(C)** of the 3D printed femur with orientation guides. Caudocranial image **(E)** of the reduced femur with the help of the 3DPAG and the pre-contoured plate.

Digital imaging and communications in medicine (DICOM) files of the patient’s pelvic limbs were exported into an open-source software (3D Slicer; https://www.slicer.org), where stereolithography (STL) objects of the right and left femur were created and transferred into a computer-aided design software (Blenderfordental; https://www.blenderfordental.com). Evaluation of the affected femur alignment showed the presence of a multiplanar deformity (Figures 1C–E), with a distal femoral valgus, a caudomedial translation of 26 mm, a femoral torsion angle of 35° compared to 21° to the left, and a 34% shortening in frontal plane/ 31% in sagittal plane. The STL object of the left femur was mirrored and aligned with the distal segment of the right femur, and used as a template for virtual reduction of the affected proximal bone segment (Figure 2A). Pre-operative planning involved correction of translation, axial derotation, and distraction of the right femur. A 3.5 mm locking compression plate (LCP; DePuySynthes) of appropriate length was scanned with Aoralscan Elite (Shining 3D Technology Co., Ltd.) and an STL object was obtained. Four bicortical virtual cylinders with a diameter of 2.0 mm were placed cranially at the level of distal and proximal diaphysis, avoiding interference with the lateral plate. A Blockout tool was used to eliminate undercuts and create a passive-fit model of the affected femoral segment. Virtual orientation guides (OG) were created for the main bone segments, with an inverted virtual representation of the corresponding cortex, to achieve a “press-fit” with the guide’s contact surface (Figure 2B). Once the main fracture segments were reduced, a 3DPAG was created (Figure 2C). An additional tolerance of 0.04 mm was incorporated into the guide design to prevent pin entrapment within the guide holes. Both femora and the 3D guides were exported as STL files and transferred to the printing software (Chitubox; https://www.chitubox.com/en/index). The prints were generated using a 3D Printer (Phrozen Mighty 4 K, Phrozen) in a biocompatible and autoclavable resin (Dental Ortho Model Resin, Phrozen). Post-processing of the prints consisted in cleaning with Isopropyl Alcohol and subsequent curing with a commercial UV lamp (Phrozen Cure Luna, Phrozen). A 3.5 mm LCP plate was precontoured to the lateral aspect of the reconstructed femur, allowing placement of four screws in the proximal fragment and three screws in the distal fragment (Figures 2D,E). Finally, the prints and the plate were sterilized. The entire pre-operative planning and printing process was completed within 24 h of admission.

The following day, the patient underwent anesthesia and surgery. Premedication consisted of methadone (Comfortan; Dechra) 0.2 mg/kg IV and medetomidine (Dorbene; Zoetis) 0.005 mg/kg IV. The induction was performed with Propofol (Narcofol; CP Pharma) given IV to effect, and the anesthesia was maintained with a mixture of isoflurane (IsoFlo; Zoetis) in oxygen. Regional anesthesia was performed with lidocaine (Lidocain; B. Braun) 2 mg/kg, administered into the lumbosacral epidural space. Perioperative amoxicillin and clavulanic acid (AmoxClav; Hexal) 15 mg/kg IV was administered 30 min before surgery and every 90 min until recovery. The dog was placed in left lateral recumbency and a lateral approach to the right femur and stifle was performed. The OG were placed and temporarily fixed with 2.0 mm Steinmann pins. Freehand ostectomies were performed, to enable realignment of the fracture segments. At this point, the OG were removed, but the pins remained in place. Due to muscle contracture and fibrosis at the fracture site, a more aggressive dissection was needed for fracture reduction and 3DPAG placement. The fracture segments were reduced, so the pins could slide through the 3DPAG until a “press-fit” was achieved proximally and distally. The precontoured LCP plate was secured with four screws proximally and three screws distally. The 3DPAG and pins were removed, and the surgical wound was routinely closed.

**Figure 3:**
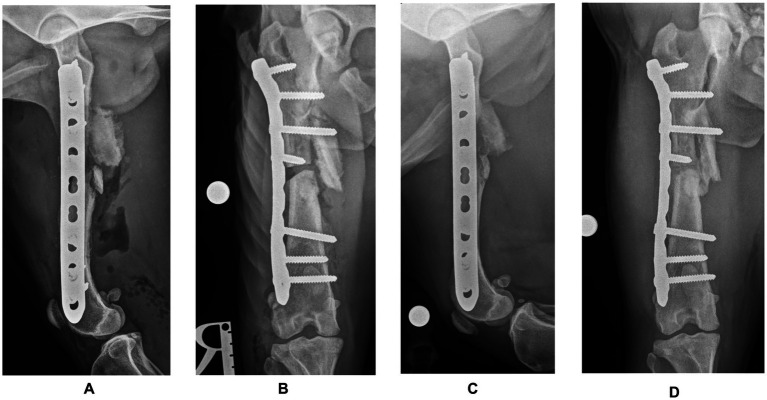
Postoperative mediolateral **(A)** and craniocaudal **(B)** radiographs of the right femur confirming satisfactory fracture alignment and implant placement. Postoperative radiographs **(C,D)** at the three-month follow-up showing almost complete healing at the ostectomy site and no signs of implant failure.

Postoperative radiographs confirmed satisfactory fracture alignment and implant placement (Figures 3A,B). The dog was hospitalized for 24 h. Analgesia consisted of methadone (Comfortan; Dechra) at 0.1–0.3 mg/kg every 4–6 h, based on the Glasgow Canine Composite Measure Pain Scale, administered for 24 h, and meloxicam (Metacam; Boehringer Ingelheim) at 0.2 mg/kg IV once daily. The following day, the dog consistently bore weight to the affected limb, exhibiting reduced but still noticeable lameness compared to the preoperative assessment. The dog was discharged on oral meloxicam at 0.1 mg/kg for 10 days. Walks were limited to 5–10 min, three times daily for six weeks.

At the six-week follow-up, the orthopedic examination revealed no evidence of lameness. Radiographic projections showed good progress toward fracture healing, with mineralized callus formation. Three months later, the orthopaedical examination was unremarkable, and the radiographic projections showed almost complete healing at the ostectomy site and no signs of implant failure (Figures 3C,D). Postoperative femoral length of the mechanical axis was 132.22 mm in the frontal plane and 130.88 mm in the sagittal plane, compared to 136.4 mm and, respectively, 130.01 mm (contralateral femur). At this point, a gradual return to normal exercise was planned for the next four weeks.

## Discussion

This case report describes the successful use of a 3DPAG to assist in the surgical management of a femoral fracture classified as a delayed union with suspected progression toward nonunion/malunion, stabilized with a locking compression plate and screws in a dog. The surgical decision was based on marked lameness, crepitus, and pain of the affected limb, along with significant femoral torsion (34°, compared to 21° on the left), well above the normal range of 19.6 ± 7.9°, which can be a contributing factor in the pathogenesis of hip dysplasia and patellar luxation (12). Additionally, the excessive limb shortening (34% in the frontal plane, and 31% in the sagittal plane) exceeds the 20% threshold that dogs can adapt to (13).

Given the limitations of CT imaging in accurately reflecting the periosteal reaction and fibrous tissue, which could result in improper placement of a custom cutting guide (14), it was decided to perform the ostectomy freehand.

The pre-contoured LCP plate allowed in this case a secure fixation with four screws placed proximally and three distally. Due to the lack of medial cortical bone in the distal aspect of the proximal fragment, only the two most proximal screws were bicortical. This configuration achieved, however, the minimum of six cortices per fragment, as recommended by Arbeitsgemeinschaft für Osteosynthesefragen (AO; Association for the Study of Internal Fixation) (15). While post-operative radiographs showed successful alignment and implant placement, a post-op CT would have been ideal to further confirm the alignment accuracy and 3DPAG precision. Nevertheless, the postoperative radiographs obtained showed a minimal femoral length shortening of 4 mm (2.93%) in the frontal plane and 0.87 mm (0.67%) in the sagittal plane, compared to the contralateral femur. No signs of varus/valgus or procurvatum/recurvatum were noted on the radiographs. Follow-up radiographs revealed good progress toward bone healing, with healthy callus formation and no changes in implant placement or alignment. The dog was able to bear weight and walk with minimal lameness just one day after surgery and three months later, there were no visible signs of lameness.

Similar 3DPAGs and 3D-printed bone models have been used to treat both ALDs and comminuted fractures in dogs and cats (7–10, 16–18). While Muroi et al. (18) and Pavarotti and Boudrieau (9) demonstrated the efficacy of using 3D-printed bone models to correct femoral malunions, this case also combined both 3D-printed bone models and 3DPAG.

Alignment guides have previously been used for corrective osteotomies such as distal femoral varus and torsional deformities (16), and in a malunion that was stabilized with an interlocking nail (10), by comparison, this case report applied 3DPAG for a delayed union/nonunion/malunion femoral fracture, reduced and stabilized with a different apparatus (plate and screws). Another case, reported by Lee et al. (11), involving a 3D-printed titanium scaffold, rh-BMP, and double plating, highlights the potential of custom 3D-printing in complex femoral reconstructions. However, their approach aimed at structural replacement and biological enhancement, while our case demonstrates the use of 3DPAG to achieve precise realignment and stabilization.

## Conclusion

This case report demonstrates the potential of 3DPAG technology in achieving accurate fracture reduction in challenging clinical scenarios such as delayed union, nonunion or malunion cases. While further refinements in guide placement techniques are warranted to enhance its clinical applicability, this approach offers a promising avenue for improved fracture healing outcomes.

## Data Availability

The raw data supporting the conclusions of this article will be made available by the authors, without undue reservation.
